# Low adherence to recommended use of neoadjuvant chemotherapy for muscle-invasive bladder cancer

**DOI:** 10.1007/s00345-023-04443-7

**Published:** 2023-05-31

**Authors:** Lisa M. C. van Hoogstraten, Calvin C. O. Man, J. Alfred Witjes, Richard P. Meijer, Sasja F. Mulder, Tineke J. Smilde, Theodora M. Ripping, Lambertus A. Kiemeney, Katja K. H. Aben, BlaZIB Study Group, J. Alfred Witjes, Theodora M. Ripping, Joost L. Boormans, Catharina A. Goossens-Laan, Antoine G. van der Heijden, Michiel S. van der Heijden, Sipke Helder, Tom J. N. Hermans, Maarten C. C. M. Hulshof, Anna M. Leliveld, Geert J. L. H. van Leenders, Richard P. Meijer, Reindert J. A. van Moorselaar, Juus L. Noteboom, Jorg R. Oddens, Theo M. de Reijke, Bas W. G. van Rhijn, Joep G. H. van Roermund, Guus W. J. Venderbosch, Bart P. Wijsman

**Affiliations:** 1 Netherlands Comprehensive Cancer Organisation, PO Box 1281, 6501 BG Nijmegen, The Netherlands; 2 Radboud Institute for Health Sciences, Radboud University Medical Centre, Nijmegen, The Netherlands; 3 Department of Urology, Radboud University Medical Centre, Nijmegen, The Netherlands; 4 Department of Oncological Urology, University Medical Centre Utrecht, Utrecht, The Netherlands; 5 Department of Medical Oncology, Radboud University Medical Centre, Nijmegen, The Netherlands; 6 Department of Medical Oncology, Jeroen Bosch Hospital, ‘s-Hertogenbosch, The Netherlands

**Keywords:** Bladder carcinoma, Guideline adherence, MIBC, Muscle-invasive bladder cancer, Neoadjuvant chemotherapy, Radical cystectomy, Variation in healthcare

## Abstract

**Purpose:**

To evaluate guideline adherence and variation in the recommended use of neoadjuvant chemotherapy (NAC) and the effects of this variation on survival in patients with non-metastatic muscle-invasive bladder cancer (MIBC).

**Patients and methods:**

In this nationwide, Netherlands Cancer Registry-based study, we identified 1025 patients newly diagnosed with non-metastatic MIBC between November 2017 and November 2019 who underwent radical cystectomy. Patients with ECOG performance status 0–1 and creatinine clearance ≥ 50 mL/min/1.73 m^2^ were considered NAC-eligible. Interhospital variation was assessed using case-mix adjusted multilevel analysis. A Cox proportional hazards model was used to evaluate the association between hospital specific probability of using NAC and survival. All analyses were stratified by disease stage (cT2 versus cT3-4a).

**Results:**

In total, of 809 NAC-eligible patients, only 34% (n = 277) received NAC. Guideline adherence for NAC in cT2 was 26% versus 55% in cT3-4a disease. Interhospital variation was 7–57% and 31–62%, respectively. A higher hospital specific probability of NAC might be associated with a better survival, but results were not statistically significant (HR_cT2_ = 0.59, 95% CI 0.33–1.05 and HR_cT3-4a_ = 0.71, 95% CI 0.25–2.04).

**Conclusion:**

Guideline adherence regarding NAC use is low and interhospital variation is large, especially for patients with cT2-disease. Although not significant, our data suggest that survival of patients diagnosed in hospitals more inclined to give NAC might be better. Further research is warranted to elucidate the underlying mechanism. As literature clearly shows the potential survival benefit of NAC in patients with cT3-4a disease, better guideline adherence might be pursued.

**Supplementary Information:**

The online version contains supplementary material available at 10.1007/s00345-023-04443-7.

## Introduction

European guidelines recommend cisplatin-based neoadjuvant chemotherapy (NAC) preceding radical cystectomy (RC) in cisplatin-eligible patients with non-metastatic muscle-invasive bladder cancer (MIBC) [1]. This recommendation is based on meta-analyses showing a significant absolute 5-year survival benefit of 5–9% in favor of NAC compared to upfront RC [2–5]. Despite this recommendation, NAC administration rates vary largely in clinical practice [6–8]. This variation might, in part, be explained by more recent studies and meta-analyses showing contradicting results regarding the benefit of NAC [9, 10]. The meta-analysis by Hamid et al. evaluated overall survival (OS) in 17 randomized controlled trials (RCTs) and retrospective studies up to 2020, and found a significant survival benefit in favor of NAC; the pooled hazard ratio (HR) for OS was 0.82 (95% CI 0.71–0.95). In contrast, the RCT-based meta-analysis by Li et al. showed no convincing evidence in favor of NAC: HR for OS was 0.92 (95% CI 0.84–1.00) and HR = 0.95 (95% CI 0.69–1.29) for progression-free survival, although the latter endpoint was only evaluated in 6 of the 14 included studies. A recent population-based observational study performed in the Netherlands including 5517 patients showed no significant survival benefit of NAC in patients with cT2N0M0 bladder cancer in contrast with cT3-4aN0M0 bladder cancer[11], suggesting to reevaluate the use of NAC in patients with cT2-disease.

In the Netherlands, the NAC utilization rate for MIBC increased from 0.6% in 1995 to 21% in 2013 [7] and is still increasing [12]. Variation in NAC use in current clinical practice is expected but underlying factors are largely unknown, as is the effect on outcome. This study aims to evaluate guideline adherence and variation in NAC use and to gain insight in the factors associated with use of NAC, taking patient eligibility into account, and to assess the effect of interhospital variation in use of NAC on survival.

## Patients and methods

This study is part of the nationwide, prospective BlaZIB study, aiming to provide insight and eventually improve the quality of bladder cancer care in the Netherlands. Details of the BlaZIB protocol were described previously [13]. The data collection of BlaZIB is embedded in the Netherlands Cancer Registry (NCR), hosted by the Netherlands Comprehensive Cancer Organisation. We selected all patients ≥ 18 years newly diagnosed with cT2–4aN0/xM0/x MIBC between 1 November 2017 and 31 October 2019 who underwent RC. A detailed description of all variables included is given in Table S1.

### Definitions

Patients were categorized into two treatment groups: NAC + RC or upfront RC. Platinum-eligibility was based on renal function and performance status. Patients were considered platinum-eligible if they had an estimated glomerular filtration rate (eGFR) ≥ 50 mL/min/1.73 m^2^ and ECOG performance score 0–1, allowing eligibility for different chemotherapeutic agents and schedules [1]. Patients were considered platinum-ineligible if eGFR < 30 mL/min/1.73 m^2^ and/or ECOG ≥ 3. The remaining patients with an eGFR between 30 and 50 mL/min/1.73 m^2^ and ECOG 0–2 were considered potentially eligible.

### Statistical analysis

Descriptive analyses were performed to evaluate guideline adherence and provide insight into patient and tumor characteristics of eligible patients, including ANOVA and Chi-square tests to evaluate differences between treatment groups. Uni- and multivariable logistic regression analyses were performed to identify factors associated with receiving NAC. Hospital-specific probabilities for eligible patients to have NAC were evaluated using multilevel logistic regression analysis, both unadjusted (i.e., observed probability) and adjusted for relevant case-mix factors. Hospitals with less than 5 observations were excluded from multilevel modelling. Two-year overall survival (OS) of patients diagnosed in hospitals with the 15% lowest and 15% highest hospital-specific probabilities of administering NAC regardless of whether patients actually received NAC was evaluated using the Kaplan Meier method and Log-Rank test. This way we gain insight in whether patients diagnosed in hospitals which were more inclined to give NAC have better outcomes compared to patients diagnosed in hospitals which were much more hesitant. Start of follow-up was defined as date of diagnosis. End of follow-up was defined as last date of follow-up or death, whatever came first. Follow-up was censored at 2 years. A Cox proportional hazards model was constructed to evaluate the effect of interhospital variation on survival, adjusted for relevant case-mix factors. All analyses were stratified by disease stage (cT2 versus cT3–4a). As a sensitivity analysis, we repeated all analyses, now including potentially NAC-eligible patients as well. Missing data were imputed using single and multiple (n = 20) imputation, assuming data being missing at random. Single imputed data were used to perform survival- and Cox regression analyses, multiple imputed data were used for all other analyses.

All statistical analyses were performed using SAS version 9.4 (SAS Institute, Cary, North Carolina, USA). P < 0.05 was considered statistically significant.

## Results

Seventy-nine percent (n = 809) of all included patients were considered NAC-eligible, but only 34% (n = 277) received NAC. Of the 180 patients considered potentially eligible, 13% (n = 23) received NAC. None of the 36 ineligible patients received NAC. Patient and tumor characteristics of eligible patients are presented in Table 1. Relatively more patients with cT3–4a disease received NAC compared to patients with cT2-disease: 55% (128 out of 233) versus 26% (149 out of 576), respectively. Most patients receiving NAC started with a multiagent, cisplatin-based regimen (95%) and had 2–4 cycles (90%). All were under 80 years of age at diagnosis. A detailed description of all 1025 patients included in this study is given in Table S2.

**Table 1:** Patient and tumor characteristics of platinum-eligible patients, diagnosed with non-metastatic muscle-invasive bladder cancer who underwent radical cystectomy, stratified by use of neoadjuvant chemotherapy (imputed data)

	All patients (N = 809)		Upfront RC (N = 532)		NAC + RC (N = 277)		P-value*
	N	(%)	N	(%)	N	(%)	
Number of administered cycles							
1	–		–		22	(7.9%)	
2	–		–		34	(12.3%)	
3	–		–		78	(28.2%)	
4	–-		–		137	(49.5%)	
5 or more	–		–		3	(1.1%)	
Unknown	–		–		3	(1.1%)	
Surgical approach							0.0553
Open	428	(52.9%)	288	(54.1%)	140	(50.5%)	
Robot-assisted	349	(43.2%)	217	(40.8%)	132	(47.7%)	
Laparoscopic, not specified	30	(3.7%)	25	(4.7%)	5	(1.8%)	
Unknown	2	(0.2%)	2	(0.4%)	0	(0.0%)	
Gender							0.0286
Male	582	(72.0%)	396	(74.5%)	186	(67.2%)	
Female	227	(28.0%)	136	(25.5%)	91	(32.8%)	
Age at diagnosis (median, IQR)	69.0	(63.0–74.0)	71.0	(65.0–76.0)	65.0	(58.0–70.0)	< 0.0001
Age at diagnosis							< 0.0001
< 60 years	143	(17.6%)	60	(11.2%)	83	(29.9%)	
60–70 years	263	(32.5%)	145	(27.3%)	118	(42.5%)	
70–80 years	349	(43.1%)	272	(51.2%)	76	(27.6%)	
≥ 80 years	55	(6.8%)	55	(10.3%)	0	(0.0%)	
Body Mass Index (BMI) (median, IQR)	26.0	(23.6–29.0)	25.9	(23.6–28.7)	26.0	(23.6–29.1)	0.1694
Body Mass Index (BMI)							0.1624
Underweight (< 18.5)	13	(1.7%)	10	(1.9%)	3	(1.2%)	
Normal weight (18.5–24.9)	308	(38.1%)	200	(37.6%)	108	(39.0%)	
Overweight (25.0–29.9)	357	(44.1%)	245	(46.1%)	111	(40.2%)	
Obese (≥ 30.0)	131	(16.1%)	77	(14.4%)	54	(19.5%)	
Weighted Charlson Comorbidity Index (CCI)							< 0.0001
0	432	(53.5%)	252	(47.4%)	180	(65.0%)	
1	233	(28.8%)	166	(31.2%)	67	(24.2%)	
2 or more	143	(17.7%)	114	(21.3%)	30	(10.8%)	
Performance status (ECOG)							0.8020
ECOG 0	575	(71.0%)	379	(71.3%)	195	(70.5%)	
ECOG 1	234	(29.0%)	153	(28.7%)	82	(29.5%)	
Renal function (eGFR) (median, IQR)	74.0	(62.1–88.0)	72.0	(61.0–86.0)	77.0	(66.0–89.3)	< 0.0001
Socioeconomic status (SES)							0.4580
Low	213	(26.3%)	143	(27.0%)	70	(25.1%)	
Middle	348	(43.0%)	233	(43.9%)	115	(41.4%)	
High	248	(30.6%)	155	(29.1%)	93	(33.5%)	
Disease stage (cTNM)							< 0.0001
cT2N0/xM0/x	576	(71.2%)	426	(80.2%)	149	(54.0%)	
cT3N0/xM0/x	205	(25.3%)	99	(18.7%)	106	(38.1%)	
cT4aN0/xM0/x	28	(3.5%)	6	(1.1%)	22	(7.9%)	
Tumor histology							0.0948
Urothelial carcinoma	788	(97.4%)	516	(97.0%)	272	(98.2%)	
Squamous cell carcinoma	6	(0.7%)	6	(1.0%)	0	(0.0%)	
Adenocarcinoma	11	(1.3%)	6	(1.1%)	5	(1.8%)	
Other	5	(0.6%)	5	(0.9%)	0	(0.0%)	
Hospital of MDTM							0.9656
Community hospital	252	(31.1%)	167	(31.4%)	85	(30.5%)	
Non-university referral hospital	420	(51.9%)	275	(51.8%)	144	(52.1%)	
University hospital	137	(17.0%)	89	(16.8%)	48	(17.3%)	

*RC *radical cystectomy, *NAC *neoadjuvant chemotherapy,* IQR *interquartile range, *ECOG *Eastern Cooperative Oncology Group,* eGFR *estimated glomerular filtration rate,* MDTM *multidisciplinary team meeting

*P-value was calculated using Chi-square for categorical variables and ANOVA for continuous variables

Multivariable regression analysis showed that increasing age (OR = 0.93, 95% CI 0.91–0.95) and presence of comorbidity (CCI ≥ 2 versus 0: OR = 0.52, 95% CI 0.31–0.88) significantly decreased the odds of having NAC in eligible patients (Table 2). Higher disease stage (cT3-4a versus cT2: OR = 3.33, 95% CI 2.36–4.71) increased the odds. Better renal function (OR = 1.02, 95% CI 1.01–1.03) and female gender (OR = 1.44, 95% CI 1.05–1.98) were univariably associated with having NAC, but these effects became non-significant in multivariable analyses. No significant associations were found for BMI, performance status, SES, tumor histology and hospital of MDTM. After stratification by disease stage, higher BMI became positively associated whereas CCI was no longer significantly associated with having NAC in patients with cT2-disease. The sensitivity analysis including both eligible and potentially eligible patients yielded similar results, except that renal function remained statistically significant in multivariable analysis (Table S3).

**Table 2:** Uni- and multivariable logistic regression analysis on the association between patient, tumor and hospital characteristics and receiving NAC, in platinum-eligible patients diagnosed with non-metastatic muscle-invasive bladder cancer who underwent radical cystectomy

	All disease stages (cT2–4a)				cT2-disease only				cT3-4a disease only			
	Univariable model		Multivariable model		Univariable model		Multivariable model		Univariable model		Multivariable model	
	OR	(95% CI)	OR	(95% CI)	OR	(95% CI)	OR	(95% CI)	OR	(95% CI)	OR	(95% CI)
**Gender**												
Male	Ref.		Ref.		Ref.				Ref.			
Female	1.44	(1.05–1.98)	1.27	(0.89–1.82)	1.42	(0.95–2.12)			1.68	(0.93–3.04)		
Age at diagnosis (per year increase)	0.92	(0.90–0.94)	0.93	(0.91–0.95)	0.91	(0.89–0.93)	0.91	(0.89–0.93)	0.94	(0.91–0.97)	0.95	(0.92–0.99)
Body Mass Index (per kg/m^2^ increase)	1.01	(0.98–1.05)			1.06	(1.01–1.11)	1.07	(1.01–1.12)	0.98	(0.92–1.04)		
Weighted Charlson Comorbidity Index												
0	Ref.		Ref.		Ref.		Ref.		Ref.		Ref.	
1	0.56	(0.40–0.80)	0.71	(0.48–1.04)	0.65	(0.41–1.01)	0.74	(0.45–1.20)	0.51	(0.27–0.96)	0.55	(0.29–1.07)
2 or more	0.37	(0.23–0.60)	0.52	(0.31–0.88)	0.49	(0.27–0.90)	0.62	(0.32–1.19)	0.24	(0.10–0.56)	0.32	(0.13–0.77)
Performance status												
ECOG 0	Ref.				Ref.				Ref.			
ECOG 1	1.04	(0.70–1.56)			1.22	(0.74–1.98)			0.86	(0.45–1.66)		
Renal function (eGFR, per mL/min/1.73 m^2^ increase)	1.02	(1.01–1.03)	1.00	(0.99–1.02)	1.01	(1.00–1.03)	1.00	(0.98–1.01)	1.03	(1.01–1.05)	1.02	(0.99–1.04)
Socio-economic status (SES)												
Low	Ref.				Ref.				Ref.			
Middle	1.02	(0.71–1.47)			1.00	(0.62–1.60)			1.30	(0.69–2.45)		
High	1.23	(0.84–1.80)			1.24	(0.75–2.03)			1.35	(0.69–2.65)		
Disease stage (cTNM)												
cT2N0/xM0/x	Ref.		Ref.									
cT3-4aN0/xM0/x	3.49	(2.54–4.80)	3.33	(2.36–4.71)								
Tumor histology												
Urothelial carcinoma	Ref.				Ref.				Ref.			
Squamous cell carcinoma	–				–				–			
Adenocarcinoma	–				–				–			
Small cell carcinoma	1.58	(0.48–5.24)			1.92	(0.53–6.89)			–			
Other	–				–				–			
Hospital of MDTM												
Community hospital	Ref.				Ref.				Ref.			
Non-university referral hospital	1.04	(0.75–1.45)			1.01	(0.67–1.52)			0.96	(0.50–1.85)		
University hospital	1.06	(0.68–1.64)			1.01	(0.55–1.87)			0.54	(0.27–1.12)		

*NAC *neoadjuvant chemotherapy,* ECOG *Eastern Cooperative Oncology Group,* eGFR *estimated glomerular filtration rate, *MDTM *multidisciplinary team meeting

The multivariable model for all disease stages included gender, age at diagnosis, weighted Charlson Comorbidity Index, renal function and disease stage. The multivariable model for cT2-stage included age, BMI, weighted Charlson Comorbidity Index and renal function. The multivariable model for cT3-4a stage included age, weighted Charlson Comorbidity Index and renal function

**Fig. 1:**
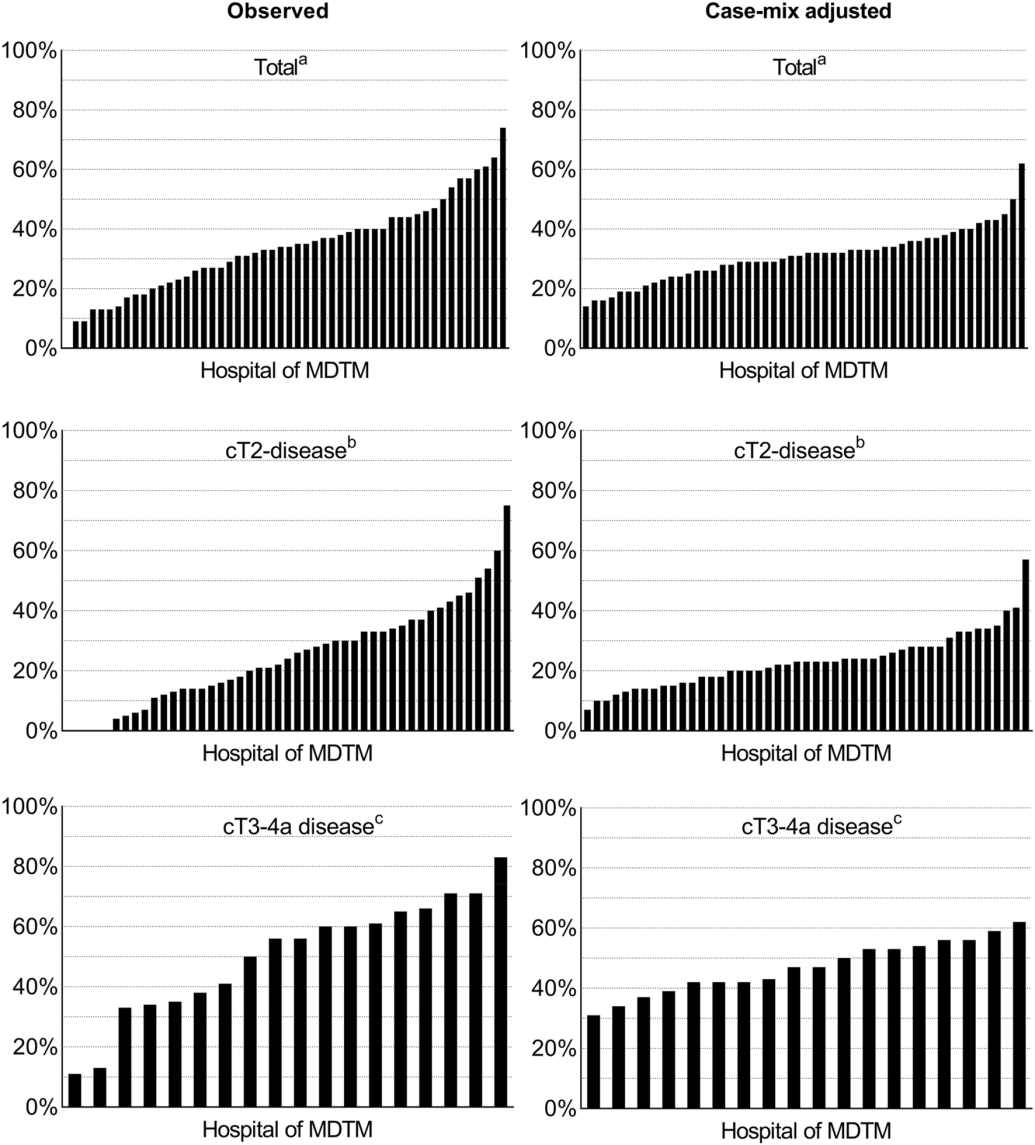
The probability of platinum-eligible patients to receive NAC per hospital* overall, for cT2-disease only and for cT3-4a disease only, observed and adjusted for case-mix factors. *NAC* neoadjuvant chemotherapy, *MDTM* multidisciplinary team meeting. *Hospitals with < 5 cases were excluded from analysis. a: The multilevel model for all disease stages (cT2–4a) included: age at diagnosis, comorbidity and disease stage, based on 52 hospitals; b: the multilevel model for cT2-disease only included: age at diagnosis and BMI, based on 47 hospitals; c: the multilevel model for cT3-4a disease only included: age at diagnosis and comorbidity, based on 18 hospitals

Large variation was observed in hospital-specific probabilities to administer NAC in platinum-eligible patients, which was 14–62% after correction for case-mix factors, i.e., age at diagnosis, comorbidity and disease stage (Fig. 1). Stratification by disease stage revealed considerable differences in NAC administration probabilities; 7–57% for patients with cT2-stage and 31–62% for patients with cT3-4a stage.

Unadjusted 2-year OS was 79% for patients diagnosed in hospitals with high probability of administering NAC and 68% for patients diagnosed in hospitals with low probability (Log-Rank test p = 0.07, Fig. S1a). This is regardless of whether patients actually received NAC or not. Stratified analysis by disease stage showed a 2-year OS of 81% versus 64% in cT2-disease (p = 0.03, Figure S1b), and 66% versus 62% in cT3-4a disease (p = 0.53, Figure S1c). Cox regression analysis in patients with T2-disease, adjusted for age at diagnosis and BMI resulted in a hazard ratio of HR_cT2_ = 0.59 (95% CI 0.33–1.05) and HR_cT3-4a_ was 0.71 (95% CI 0.25–2.04) in patients with T3-4a disease, adjusted for age at diagnosis and comorbidity (Table S4).

## Discussion

In this nationwide, population-based study, we evaluated guideline adherence and variation in the recommended use of neoadjuvant chemotherapy preceding radical cystectomy as curative treatment for MIBC. We found that guideline adherence was fairly low, i.e., only 26% for cT2- and 55% for cT3-4a disease. Factors associated with NAC were age at diagnosis, comorbidity and disease stage. Large interhospital variation in NAC use was observed, especially for patients with cT2-disease, for whom 2-year overall survival appeared to be better for those diagnosed in hospitals with high probability of administering NAC compared to hospitals with a low probability.

This study showed that the minority of platinum-eligible patients actually received NAC. Reasons to abstain from NAC, as noted in the medical files, were among others the patients’ preference, limited expected survival gain, patients’ age and functional status, and presence of hearing loss. These patients, except for ten, also did not receive any adjuvant chemotherapy (*data not shown*). Although for two-thirds of patients no reason was documented for not receiving NAC, these results indicate there are more factors in play than those considered in the eligibility criteria alone.

Patients with younger age, no comorbid conditions and/or cT3/cT4a bladder cancer received NAC more often, which was expected and is in line with previous studies [6, 14]. Patients who underwent upfront RC had lower renal function compared to patients treated with NAC + RC, but we anticipated an even lower mean renal function for patients undergoing upfront RC. It is likely that patients with pre-existing renal insufficiency also suffer from (higher) comorbidity, and were, therefore, precluded from NAC and did not even undergo RC at all. Despite being eligible, age remained statistically significant in our multivariable regression analysis after correction for renal function, comorbidity and disease stage, indicating that older patients are less often offered NAC or may decline NAC more often compared to younger patients. Multiple studies, reviews and even international guidelines state that, next to patient preferences, not chronological but biological age (i.e., organ function, comorbidity, frailty and functional status) should be taken into account in treatment decision-making [1, 15, 16].Therefore, it might be unjustified that chronological age plays such a prominent role in clinical practice.

We observed low and varying guideline adherence between hospitals. This is in agreement with previous studies demonstrating low NAC utilization rates in cisplatin-eligible patients, varying from 12 to 31% [8, 17, 18]. Substantial variation remained after case-mix adjustment, especially for patients with cT2-disease (7–57%), indicating that hospital/doctor factors likely play a role in the use of NAC. An explanation would be that hospitals follow their own institutional and/or regional guideline agreements in addition to the European guidelines. Within our BlaZIB study, a survey was conducted among urologists regarding institutional NAC-practice patterns. The survey revealed that, although recommended in international guidelines, 9 out of 70 included hospitals do not offer NAC to patients with cT2-disease by default, possibly due to the limited survival benefit of NAC for cT2-disease shown in several studies. In fact, the meta-analyses on which the recommendation concerning NAC was based, included two large RCTs, i.e. the Nordic Cystectomy Trials I and II [19, 20], that failed to show survival benefit in favor of NAC for cT2N0M0 compared to cT3-4aN0M0 bladder cancer. Two other trials, i.e., the MRC/EORTC trial and trial BA06-30894, did not perform stage-specific analyses [21, 22]. A US study comparing real-world data of 8732 patients with cT2–4aN0M0 bladder cancer who underwent RC between 2004 and 2012 to the results of the SWOG-8710 trial found no survival advantage of NAC either [23]. The authors attributed their findings to important differences between baseline characteristics of patients in clinical studies and those treated in general clinical practice. It is likely that utilization and efficacy of NAC are lower in real life compared to clinical studies. In that case, patients might experience no beneficial or even worse outcomes compared to patients undergoing upfront RC, since time to RC is prolonged when administering NAC. Further research is recommended to address the real-life efficacy of NAC in patients with cT2-disease.

For patients with cT3-4a disease, case-mix adjusted interhospital variation was slightly smaller. Nevertheless, our results suggest there is room for improvement regarding the use and guideline adherence of NAC in these patients. The attitude of physicians towards NAC is fundamental for its use, as believers in NAC are more likely to recommend NAC [24], and patients tend to follow recommendations from their doctor [25].

The large interhospital variation in NAC use did not significantly impact overall survival. However, there appears to be a trend in favor of hospitals with higher probability of administering NAC. For both cT2 and cT3-4a disease, these hospitals appeared to perform better regarding survival compared to hospitals with low probability, regardless of whether patients actually received NAC. This finding suggests factors other than NAC itself are important. Hospitals with higher NAC probability might have higher patient volumes, more surgical experience and more expertise on bladder cancer, resulting in better patient selection for specific treatment and better surgical outcomes affecting survival. Hospitals with the highest probability of administering NAC indeed appear to have a slightly higher patient volume (*data not shown*), but more research is needed to elucidate the underlying mechanisms.

In this study, we provided detailed insight into the variation in NAC use, the factors associated with receiving NAC, and whether patient outcomes were better if patients were diagnosed in hospitals that are more inclined to give NAC compared to more hesitant hospitals, taking eligibility into account. However, the observational study design has to be recognized as a limitation. Missing values, often arising from poor documentation in the electronic medical files, are inherent to this design and were addressed by employing imputation. To check the robustness of our results after imputation on performance status, we performed a sensitivity analyses repeating our analyses; once assuming that all patients with missing performance status have an ECOG score of 0 and once assuming they have an ECOG score of 3 as this will affect the number of patients considered eligible. Our results remained fairly similar, indicating that our analysis were likely to be robust (*data not shown*). If patients abstained from NAC, underlying reasons were poorly documented. Eligible patients who did not undergo NAC may have declined NAC owing to poor quality of life or other personal reasons, but we would not expect such a large difference in patients’ preferences between hospitals to fully explain the variation remaining after case-mix adjustment. We selected all patients who underwent RC, which might have led to underestimation of current guideline adherence since we could have missed patients who received NAC, but did not continue to RC. Our survival analyses might be prone to immortal time bias, but since patients planning to undergo RC are generally quite fit, we estimate the effect to be minimal. Also, using date of RC instead of date of diagnosis did not alter our results significantly (*data not shown*). Shortly after the end of the inclusion period of our study the COVID-19 pandemic emerged, disrupting regular health care. The COVID-pandemic might have affected NAC use, since use of (neoadjuvant) chemotherapy was temporarily discouraged due to potential immunosuppressive effects. To evaluate the use of NAC in more recent years post-COVID, the current study may be repeated in a few years.

In conclusion, guideline adherence regarding the recommended use of NAC is low and interhospital variation is large, especially in cT2 bladder cancer. Patients diagnosed in hospitals more likely to give NAC appear to have better case-mix adjusted survival compared to patients in hospitals with low probability, although the reported associations were not statistically significant. The underlying mechanism for this is currently unknown, further research is warranted to provide more insight. Guideline adherence in cT3-4a disease is better, but could be improved, especially as for these patients literature is consistent concerning the beneficial effect of NAC. Raising awareness amongst physicians may lead to more consistent NAC utilization between hospitals, prevent over- and undertreatment with NAC, and potentially enhance quality of life and oncological outcomes such as survival.

## Supplementary Information

Below is the link to the electronic supplementary material.

Supplementary file1 (DOCX 968 KB)

## Data Availability

All data used for this study can be requested from the NCR. All data requests are reviewed by the supervisory committee of the NCR for compliance with the NCR objectives and (inter)national (privacy) regulation and legislation (https://iknl.nl/en/ncr/apply-for-data) https://iknl.nl/en/ncr/apply-for-data.
