# Supplementary material for: Low adherence to recommended use of neoadjuvant chemotherapy for muscle-invasive bladder cancer
Source: World J Urol. 2023 May 31;41(7):1837–45. doi: 10.1007/s00345-023-04443-7 (PMC10231297; doi:10.1007/s00345-023-04443-7)
Supplement: Supplementary file 1 — Supplementary file1 (DOCX 968 KB) [file 345_2023_4443_MOESM1_ESM.docx]

**Supplementary tables and figures**

Table S1: Detailed description of the variables included in this study

| **Variables in the Netherlands Cancer Registry (NCR)** | |  |
| --- | --- | --- |
| Gender | Male or female | |
| Age at diagnosis | Categorized into <60 years, 60-70 years, 70-80 years and ≥80 years | |
| Socio-economic status | Derived from statistics Netherlands (CBS) based on the patients’ full six-digit postal code and categorized into low, middle and high | |
| Disease stage | Defined according to the 8^th^ edition of the tumour, node and metastasis (TNM) classification [1]. Clinical staging was based on physical examination, findings at cystoscopy and TURBT, computed tomography (CT-)scan of the abdomen/pelvis and chest imaging (at least a chest X-ray) | |
| Tumor histology | Defined according to the International Classification of Diseases for Oncology [2] and categorized into urothelial carcinoma, squamous cell carcinoma, adenocarcinoma and other | |
| Treatment type, start and end date | Categorized into neoadjuvant chemotherapy (NAC) + radical cystectomy (RC) or upfront RC. NAC was defined as any systemic chemotherapy administered after bladder cancer diagnosis and before RC | |
| Type of hospital | Categorized into community, non-university referral and university hospital | |
| Vital status and date of death | Obtained through annual linkage with the Personal Records Database | |
| **Additional variables in the BlaZIB study** | |  |
| Performance status | Defined according to the Eastern Cooperative Oncology Group (ECOG) performance score [3] | |
| Comorbidity | Defined according to the 1987 weighted Charlson Comorbidity Index (CCI) score [4] and categorized into 0, 1 and 2 or more | |
| Renal function | Presented as the estimated glomerular filtration rate (eGFR) in mL/min/1.73m^2^, measured before first systemic treatment | |
| Body mass index | Defined as kg/m^2^, categorized into underweight (< 18.5 kg/m^2^), normal weight (18.5-24.9 kg/m^2^), overweight (25.0-29.9 kg/m^2^) and obese (≥30.0 kg/m^2^) | |

[1] Brierley JD, Gospodarowicz MK, Wittekind C. TNM classification of malignant tumours: John Wiley & Sons; 2017.

[2] Fritz A, Percy C, Jack A, Shanmugaratnam K, Sobin LH, Parkin DM, et al. International classification of diseases for oncology. 3rd ed. Geneva: World Health Organization; 2000.

[3] Oken MM, Creech RH, Tormey DC, Horton J, Davis TE, McFadden ET, et al. Toxicity and response criteria of the Eastern Cooperative Oncology Group. Am J Clin Oncol. 1982;5(6):649-55.

[4]Charlson ME, Pompei P, Ales KL, MacKenzie CR. A new method of classifying prognostic comorbidity in longitudinal studies: development and validation. J Chronic Dis. 1987;40(5):373-83.

Table S2: Patient and tumor characteristics of all patients diagnosed with non-metastatic muscle-invasive bladder cancer who underwent radical cystectomy, stratified by use of neoadjuvant chemotherapy

|  |  | **All patients (N=1025)** | | **Upfront RC (N=725)** | | | | **NAC + RC (N=300)** | | |  |
| --- | --- | --- | --- | --- | --- | --- | --- | --- | --- | --- | --- |
|  |  | **N** | **(%)** | **N** | | **(%)** | | **N** | **(%)** | |  |
| **Gender** | |  |  |  | |  | |  |  | |  |
|  | Male | 738 | (72.0%) | 534 | | (73.7%) | | 204 | (68.0%) | |  |
|  | Female | 287 | (28.0%) | 191 | | (26.3%) | | 96 | (32.0%) | |  |
| **Age at diagnosis (median, IQR)** | | 70.0 | (63.0-75.0) | 71.0 | | (66.0-76.0) | | 65.0 | (58.0-70.0) | |  |
| **Age at diagnosis** | |  |  |  | |  | |  |  | |  |
|  | <60 years | 164 | (16.0%) | 76 | | (10.5%) | | 88 | (29.3%) | |  |
|  | 60-70 years | 326 | (31.8%) | 199 | | (27.4%) | | 127 | (42.3%) | |  |
|  | 70-80 years | 457 | (44.6%) | 372 | | (51.3%) | | 85 | (28.3%) | |  |
|  | ≥80 years | 78 | (7.6%) | 78 | | (10.8%) | | 0 | (0.0%) | |  |
| **Body Mass Index (BMI) (median, IQR)**  (Missing %) | | 26.2 | (23.7-29.1)  (5.0%) | 26.2 | | (23.7-29.0)  (4.4%) | | 26.0 | (23.7-29.1)  (6.3%) | |  |
| **Body Mass Index (BMI)** | |  |  |  | |  | |  |  | |  |
|  | Underweight (< 18.5) | 15 | (1.5%) | 12 | | (1.7%) | | 3 | (1.0%) | |  |
|  | Normal weight (18.5-24.9) | 366 | (35.7%) | 254 | | (35.0%) | | 112 | (37.3%) | |  |
|  | Overweight (25.0-29.9) | 424 | (41.4%) | 313 | | (43.2%) | | 111 | (37.0%) | |  |
|  | Obese (≥30.0) | 169 | (16.5%) | 114 | | (15.7%) | | 55 | (18.3%) | |  |
|  | Unknown | 51 | (5.0%) | 32 | | (4.4%) | | 19 | (6.3%) | |  |
| **Weighted Charlson Comorbidity Index (CCI)** | | | | |  | |  |  | |  | |
|  | 0 | 474 | (46.2%) | 295 | | (40.7%) | | 179 | (59.7%) | |  |
|  | 1 | 271 | (26.4%) | 205 | | (28.3%) | | 66 | (22.0%) | |  |
|  | 2 or more | 215 | (21.0%) | 186 | | (25.7%) | | 29 | (9.7%) | |  |
|  | Unknown | 65 | (6.3%) | 39 | | (5.4%) | | 26 | (8.7%) | |  |
| **Performance status (ECOG)** | |  |  |  | |  | |  |  | |  |
|  | ECOG 0 | 439 | (42.8%) | 265 | | (36.6%) | | 174 | (58.0%) | |  |
|  | ECOG 1 | 192 | (18.7%) | 117 | | (16.1%) | | 75 | (25.0%) | |  |
|  | ECOG 2 | 16 | (1.6%) | 13 | | (1.8%) | | 3 | (1.0%) | |  |
|  | ECOG 3 or higher | 4 | (0.4%) | 4 | | (0.6%) | | 0 | (0.0%) | |  |
|  | Unknown | 374 | (36.5%) | 326 | | (45.0%) | | 48 | (16.0%) | |  |
| **Renal function (eGFR) (median, IQR)**  (Missing %) | | 69.0 | (56.0-85.0)  (10.3%) | 67.0 | | (51.0-83.0)  (13.8%) | | 76.0 | (62.0-89.0)  (2.0%) | |  |
| **Renal function (eGFR)** | |  |  |  | |  | |  |  | |  |
|  | ≥50 mL/min/1.73m^2^ | 760 | (74.1%) | 485 | | (66.9%) | | 275 | (91.7%) | |  |
|  | 30-50 mL/min/1.73m^2^ | 137 | (13.4%) | 118 | | (16.3%) | | 19 | (6.3%) | |  |
|  | <30 mL/min/1.73m^2^ | 22 | (2.1%) | 22 | | (3.0%) | | 0 | (0.0%) | |  |
|  | Unknown | 106 | (10.3%) | 100 | | (13.8%) | | 6 | (2.0%) | |  |
| **Socioeconomic status (SES)** | |  |  |  | |  | |  |  | |  |
|  | Low | 275 | (26.8%) | 201 | | (27.7%) | | 74 | (24.7%) | |  |
|  | Middle | 434 | (42.3%) | 310 | | (42.8%) | | 124 | (41.3%) | |  |
|  | High | 316 | (30.8%) | 214 | | (29.5%) | | 102 | (34.0%) | |  |
| **Disease stage (cTNM)** | |  |  |  | |  | |  |  | |  |
|  | cT2N0/xM0/x | 709 | (69.2%) | 552 | | (76.1%) | | 157 | (52.3%) | |  |
|  | cT3N0/xM0/x | 263 | (25.7%) | 148 | | (20.4%) | | 115 | (38.3%) | |  |
|  | cT4aN0/xM0/x | 53 | (5.2%) | 25 | | (3.4%) | | 28 | (9.3%) | |  |
| **Tumor histology** | |  |  |  | |  | |  |  | |  |
|  | Urothelial carcinoma | 988 | (96.4%) | 694 | | (95.7%) | | 294 | (98.0%) | |  |
|  | Squamous cell carcinoma | 15 | (1.5%) | 14 | | (1.9%) | | 1 | (0.3%) | |  |
|  | Adenocarcinoma | 1 | (0.1%) | 1 | | (0.1%) | | 0 | (0.0%) | |  |
|  | Small cell carcinoma | 15 | (1.5%) | 10 | | (1.4%) | | 5 | (1.7%) | |  |
|  | Other | 6 | (0.6%) | 6 | | (0.8%) | | 0 | (0.0%) | |  |
| **Platinum-eligibility*** | |  |  |  | |  | |  |  | |  |
|  | Not eligible | 26 | (2.5%) | 26 | | (3.6%) | | 0 | (0.0%) | |  |
|  | Eligible | 484 | (47.2%) | 254 | | (35.0%) | | 230 | (76.7%) | |  |
|  | Potentially eligible | 97 | (9.5%) | 81 | | (11.2%) | | 16 | (5.3%) | |  |
|  | Unknown | 418 | (40.8%) | 364 | | (50.2%) | | 54 | (18.0%) | |  |
| **Chemotherapeutic agent NAC** | |  |  |  | |  | |  |  | |  |
|  | Cisplatin-based** | - |  | - | |  | | 277 | (92.3%) | |  |
|  | Carboplatin-based | - |  | - | |  | | 21 | (7.0%) | |  |
|  | Other | - |  | - | |  | | 2 | (0.7%) | |  |
| **Hospital of MDTM** | |  |  |  | |  | |  |  | |  |
|  | Community hospital | 309 | (30.1%) | 217 | | (29.9%) | | 92 | (30.7%) | |  |
|  | Non-university referral hospital | 543 | (53.0%) | 385 | | (53.1%) | | 158 | (52.7%) | |  |
|  | University hospital | 173 | (16.9%) | 123 | | (17.0%) | | 50 | (16.7%) | |  |

*RC: radical cystectomy; NAC: neoadjuvant chemotherapy; IQR: interquartile range; ECOG: Eastern Cooperative Oncology Group; eGFR: estimated glomerular filtration rate; MDTM: multidisciplinary team meeting*

* Patients were considered platinum-ineligible if they had eGFR <30 mL/min/1.73m^2^ and/or ECOG ≥3. Patients were considered platinum-eligible in case of eGFR ≥50 mL/min/1.73m^2^ and ECOG 0-1. We considered patients with eGFR 30-50 mL/min/1.73m^2^ and ECOG 0-2 potentially eligible.

**N=13 patients later switched from cisplatin to carboplatin

Table S3: Uni- and multivariable logistic regression analysis on the association between patient, tumor and hospital characteristics and receiving NAC, in platinum-eligible patients and patients with potential eligibility* diagnosed with non-metastatic muscle-invasive bladder cancer who underwent radical cystectomy

|  | **Total** | | | | **cT2-disease only** | | | | **cT3-4a disease only** | | | |
| --- | --- | --- | --- | --- | --- | --- | --- | --- | --- | --- | --- | --- |
|  | **Univariable model** | | **Multivariable model** | | **Univariable model** | | **Multivariable model** | | **Univariable**  **model** | | **Multivariable model** | |
|  | **OR** | **95% CI** | **OR** | **95% CI** | **OR** | **95% CI** | **OR** | **95% CI** | **OR** | **95% CI** | **OR** | **95% CI** |
| **Gender** |  |  |  |  |  |  |  |  |  |  |  |  |
| Male | ref. |  | ref. |  | ref. |  | ref. |  | ref. |  |  |  |
| Female | 1.39 | (1.03 - 1.87) | 1.19 | (0.85 - 1.66) | 1.49 | (1.01 - 2.20) | 1.43 | (0.94 - 2.18) | 1.16 | (0.71 - 1.91) |  |  |
| **Age at diagnosis (per year increase)** | 0.92 | (0.90 - 0.93) | 0.93 | (0.91 - 0.95) | 0.91 | (0.89 - 0.93) | 0.92 | (0.90 - 0.94) | 0.93 | (0.91 - 0.96) | 0.96 | (0.93 - 0.99) |
| **Body Mass Index (per kg/m² increase)** | 1.01 | (0.97 - 1.04) |  |  | 1.05 | (1.00 - 1.10) | 1.07 | (1.01 - 1.12) | 0.97 | (0.92 - 1.03) |  |  |
| **Weighted Charlson Comorbidity Index** | | |  |  |  |  |  |  |  |  |  |  |
| 0 | ref. |  | ref. |  | ref. |  | ref. |  | ref. |  | ref. |  |
| 1 | 0.54 | (0.39 - 0.75) | 0.66 | (0.46 - 0.94) | 0.64 | (0.41 - 0.98) | 0.72 | (0.45 - 1.15) | 0.42 | (0.24 - 0.74) | 0.51 |  |
| 2 or more | 0.28 | (0.18 - 0.44) | 0.42 | (0.26 - 0.68) | 0.37 | (0.21 - 0.65) | 0.50 | (0.27 - 0.90) | 0.19 | (0.09 - 0.41) | 0.28 | (0.12 - 0.62) |
| **Performance status** |  |  |  |  |  |  |  |  |  |  |  |  |
| ECOG 0 | ref. |  |  |  | ref. |  |  |  | ref. |  |  |  |
| ECOG 1 | 0.99 | (0.68 - 1.42) |  |  | 1.14 | (0.72 - 1.81) |  |  | 0.81 | (0.46 - 1.40) |  |  |
| ECOG 2 | 0.33 | (0.10 - 1.11) |  |  | 0.46 | (0.10 - 2.08) |  |  | 0.19 | (0.02 - 1.70) |  |  |
| **Renal function (eGFR, per mL/min/1.73m2 increase)** | 1.03 | (1.02 - 1.03) | 1.02 | (1.01 - 1.03) | 1.02 | (1.01 - 1.03) | 1.01 | (1.00 - 1.02) | 1.03 | (1.02 - 1.05) | 1.03 | (1.01 - 1.04) |
| **Socio-economic status (SES)** |  |  |  |  |  |  |  |  |  |  |  |  |
| Low | ref. |  |  |  | ref. |  |  |  | ref. |  |  |  |
| Middle | 1.12 | (0.80 - 1.58) |  |  | 1.09 | (0.69 - 1.71) |  |  | 1.36 | (0.79 - 2.36) |  |  |
| High | 1.31 | (0.92 - 1.88) |  |  | 1.31 | (0.82 - 2.10) |  |  | 1.53 | (0.85 - 2.75) |  |  |
| **Disease stage (cTNM)** |  |  |  |  |  |  |  |  |  |  |  |  |
| cT2N0/xM0/x | ref. |  | ref. |  |  |  |  |  |  |  |  |  |
| cT3-4aN0/xM0/x | 3.03 | (2.27 - 4.04) | 3.10 | (2.26 - 4.26) |  |  |  |  |  |  |  |  |
| **Tumor histology** |  |  |  |  |  |  |  |  |  |  |  |  |
| Urothelial carcinoma | ref. |  |  |  | ref. |  |  |  | ref. |  |  |  |
| Squamous cell carcinoma | 0.38 | (0.05 - 3.16) |  |  | - |  |  |  | 0.35 | (0.04 - 3.43) |  |  |
| Adenocarcinoma | - |  |  |  | - |  |  |  | - |  |  |  |
| Small cell carcinoma | 1.62 | (0.51 - 5.16) |  |  | 2.26 | (0.63 - 8.12) |  |  | 1.06 | (0.07 - 17.06) |  |  |
| Other | - |  |  |  | - |  |  |  | - |  |  |  |
| **Hospital of MDTM** |  |  |  |  |  |  |  |  |  |  |  |  |
| Community hospital | ref. |  |  |  | ref. |  |  |  | ref. |  |  |  |
| Non-university referral hospital | 0.96 | (0.71 - 1.31) |  |  | 0.92 | (0.62 - 1.35) |  |  | 0.90 | (0.52 - 1.57) |  |  |
| University hospital | 0.97 | (0.64 - 1.47) |  |  | 0.85 | (0.47 - 1.55) |  |  | 0.65 | (0.34 - 1.24) |  |  |

*OR: odds ratio; 95%CI: 95% confidence interval; NAC: neoadjuvant chemotherapy; ECOG: Eastern Cooperative Oncology Group; eGFR: estimated glomerular filtration rate; MDTM: multidisciplinary team meeting*

* Patients were considered platinum-eligible in case of eGFR ≥50 mL/min/1.73m^2^ and ECOG 0-1. We considered patients with eGFR 30-50 mL/min/1.73m^2^ and ECOG 0-2 potentially eligible.

Figure S1: Unadjusted overall survival of platinum-eligible patients with non-metastatic MIBC who underwent radical cystectomy, diagnosed in hospitals with a high versus low hospital-specific probability to receive NAC overall (a), for cT2-stage only (b) and for cT3-4a stage only (c)





Table S4: Case-mix adjusted Cox Proportional Hazards regression analyses on the association between the hospital-specific probabilities of administering NAC and overall survival, in platinum-eligible patients and patients with potential eligibility* diagnosed with non-metastatic muscle-invasive bladder cancer who underwent radical cystectomy

|  |  | Eligible patients | | Eligible and potentially eligible patients | | |
| --- | --- | --- | --- | --- | --- | --- |
|  |  | HR | 95%CI | | HR | 95%CI |
| Total^1^ | **Hospital-specific probability to administer NAC (continuous)** | 0.99 | (0.98 - 1.00) | | 0.99 | (0.98 - 1.00) |
|  | **Hospital-specific probability to administer NAC** |  | | |  | |
|  | Lowest 15% (<21%) | Ref. |  | | Ref. |  |
|  | Highest 15% (≥40%) | 0.69 | (0.44 - 1.09) | | 0.71 | (0.48 - 1.07) |
| cT2-disease^2^ | **Hospital-specific probability to administer NAC (continuous)** | 0.99 | (0.97 - 1.00) | | 0.99 | (0.98 - 1.01) |
|  | **Hospital-specific probability to administer NAC** |  | | |  | |
|  | Lowest 15% (<14%) | Ref. |  | | Ref. |  |
|  | Highest 15% (≥33%) | 0.59 | (0.33 - 1.05) | | 0.70 | (0.42 - 1.17) |
| cT3-4a disease^3^ | **Hospital-specific probability to administer NAC (continuous)** | 1.00 | (0.96 - 1.03) | | 0.99 | (0.96 - 1.02) |
|  | **Hospital-specific probability to administer NAC** |  | | |  | |
|  | Lowest 15% (<37%) | Ref. |  | | Ref. |  |
|  | Highest 15% (≥56%) | 0.71 | (0.25 - 2.04) | | 0.52 | (0.24 - 1.15) |

*HR: hazard ratio; 95%CI: 95% confidence interval*

* Patients were considered platinum-eligible in case of eGFR ≥50 mL/min/1.73m^2^ and ECOG 0-1. We considered patients with eGFR 30-50 mL/min/1.73m^2^ and ECOG 0-2 potentially eligible.

^1^ Adjusted for age at diagnosis, comorbidity and disease stage

^2^ Adjusted for age at diagnosis and BMI

^3^ Adjusted for age at diagnosis and comorbidity
